# RNA-Sequencing Reveals Upregulation and a Beneficial Role of Autophagy in Myoblast Differentiation and Fusion

**DOI:** 10.3390/cells11223549

**Published:** 2022-11-10

**Authors:** Pengcheng Lyu, Honglin Jiang

**Affiliations:** School of Animal Sciences, Virginia Tech, Blacksburg, VA 24061, USA

**Keywords:** autophagy, differentiation, myoblast, myotube, RNA-seq

## Abstract

Myoblast differentiation is a complex process whereby the mononuclear muscle precursor cells myoblasts express skeletal-muscle-specific genes and fuse with each other to form multinucleated myotubes. The objective of this study was to identify potentially novel mechanisms that mediate myoblast differentiation. We first compared transcriptomes in C2C12 myoblasts before and 6 days after induction of myogenic differentiation by RNA-seq. This analysis identified 11,046 differentially expressed genes, of which 5615 and 5431 genes were upregulated and downregulated, respectively, from before differentiation to differentiation. Functional enrichment analyses revealed that the upregulated genes were associated with skeletal muscle contraction, autophagy, and sarcomeres while the downregulated genes were associated with ribonucleoprotein complex biogenesis, mRNA processing, ribosomes, and other biological processes or cellular components. Western blot analyses showed an increased conversion of LC3-I to LC3-II protein during myoblast differentiation, further demonstrating the upregulation of autophagy during myoblast differentiation. Blocking the autophagic flux in C2C12 cells with chloroquine inhibited the expression of skeletal-muscle-specific genes and the formation of myotubes, confirming a positive role for autophagy in myoblast differentiation and fusion.

## 1. Introduction

Skeletal muscle is the largest tissue in the body, accounting for nearly half of the body’s entire weight, and has many important functions, including contraction [1]. The basic structural units of skeletal muscle are myofibers, which are multinucleated muscle cells differentiated and fused from the muscle precursor cells myoblasts [2,3,4,5]. Significant changes occur in gene transcription during myoblast differentiation and fusion into myofibers, and these changes are regulated in the nucleus by cis-regulatory elements, including enhancers and promotors, and trans-regulatory factors, including transcription factors, co-factors, and other DNA-targeted regulatory proteins [6,7]. Four master transcription factors have been identified to regulate gene transcription during myogenesis, including myogenic factor 5 (MYF5), MYF6 (also known as MRF4), MyoD (MYOD1), and myogenin (MYOG), of which MYOG is the major transcription factor that regulates gene transcription during myoblast differentiation [8,9]. DNA methylation plays a vital role in determining the expression of genes critical for myoblast differentiation via changing the accessibility of chromatin to transcription factors or co-factors [10,11]. Within the cytoplasm, multiple signal transduction pathways, including the mTOR and AMPK pathways, ensure accurate epigenetic control of gene expression during myoblast differentiation [12,13]. Additionally, microRNA and lncRNA control the protein buildup in differentiating myoblasts partly via altering the stability of mRNA [14,15]. On the cell surface, various receptors respond to extracellular signals, such as amino acids, hormones, cytokines, and mechanical damage, and initiate the intracellular signal transduction pathways involved in myoblast differentiation [9,16,17,18].

It can be easily imagined that significant protein turnover occurs in the cytoplasm during myoblast differentiation and fusion and that myoblast differentiation and fusion might be controlled by protein degradation. Autophagy is a major intracellular protein degradation process by which unnecessary or damaged proteins or organelles are degraded and reused as raw materials to reconstruct new cellular components [19]. This process includes packaging the target objects in the phagophores derived from the endoplasmic reticulum [20]; the expansion and sealing of the phagophores with various proteins, including different Atg isoforms [21]; the establishment of autophagosomes; and the fusion of autophagosomes with lysosomes to form autolysosomes, where the target material for recycling is degraded by lysosomal hydrolases. Autophagy has been linked to muscle regeneration following injury [12], muscle aging [22], muscle atrophy [23], and myoblast differentiation [24,25,26,27,28,29].

The objective of this research was to uncover potentially novel mechanisms that regulate myoblast differentiation. Through an RNA-seq analysis of transcriptomes in C2C12 myoblasts, we identified many biological processes, including autophagy, associated with myoblast differentiation. Through biochemical and immunocytochemical analyses, we confirmed the beneficial role of autophagy-mediated protein degradation in myoblast differentiation and fusion.

## 2. Materials and Methods

### 2.1. Major Reagents Used in this Study

Growth medium (GM) was composed of 89% Dulbecco’s Modified Eagle Medium (DMEM), 10% fetal bovine serum (FBS), and 1% Antibiotic-Antimycotic (ABAM). Differentiation medium (DM) consisted of 97% DMEM, 2% horse serum, and 1% ABAM. Radioimmunoprecipitation assay (RIPA) buffer consisted of 150 mM NaCl, 5 mM ethylenediaminetetraacetic acid (EDTA), 50 mM Tris-base pH 8.0, 1% Nonidet P40, 0.5% sodium deoxycholate, 0.1% sodium dodecyl sulfate (SDS), and 1% Halt Protease Inhibitor Cocktail. Western blot running buffer consisted of 25 mM Tris-base pH 8.0, 190 mM glycine, and 3.5 mM SDS. Western blot transfer buffer consisted of 25 mM Tris-bas pH 8.0, 190 mM glycine, and 20% methanol. Tris-buffered saline with Tween 20 (TBST) buffer consisted of 20 mM Tris-bas pH 8.0, 50 mM NaCl, and 0.05% Tween20. The blocking buffer was TBST with 5% non-fat milk added.

C2C12 cells (catalog number CRL-1772) were purchased from ATCC (Manassas, VA, USA), FBS (S11150) from Atlanta Biologicals (Minneapolis, MN, USA), ABAM (30-004-CI) from Corning (Corning, NY, USA), DMEM (11965118) from Thermo Fisher Scientific (Waltham, MA, USA), non-fat milk (M0841) from Labscientific (Highlands, NJ, USA), chloroquine (14194) from Cayman (Ann Arbor, MI, USA), TRIzol Reagent (15596026) from Invitrogen (Waltham, MA, USA), a Direct-zol RNA Microprep kit (R2062) from Zymo Research (Irvine, CA, USA), and nitrocellulose membranes (1620147) from BIO-RAD (Hercules, CA, USA). Random primers (C1181), ImProm-II Reverse Transcriptase (A3803), RNasin Ribonuclease Inhibitor (N2615), and Deoxyribonucleoside Triphosphate (dNTP) Mix (U1515) were purchased from Promega (Madison, WI, USA). Fast SYBR Green Master Mix (4385612) was purchased from Applied Biosystems (Waltham, MA, USA). The NEBNext Ultra II Directional RNA Library Prep Kit for Illumina (E7760S) was purchased from New England BioLabs (Ipswich, MA, USA). All other reagents and materials used in this study were purchased from either Thermo Fisher Scientific or Sigma-Aldrich (St. Louis, MO, USA), unless otherwise indicated.

### 2.2. Cell Culture

C2C12 cells were cultured in GM to expand. When they reached approximately 70% confluency, they were split into 6-well plates at a density of 5 × 10^5^ cells/well. After one-day culture in GM or when they reached more than 90% confluency, GM was replaced with DM to induce myogenic differentiation, as described previously [30]. To block autophagy in C2C12 cells, 10 μM or 20 μM chloroquine, which inhibits autophagy by blocking the formation of autolysosomes from autophagosomes and lysosomes [31,32,33], was added to the culture medium. Pilot experiments showed that at concentrations higher than 20 μM, chloroquine caused significant death in C2C12 cells. To control wells 0.1% water was added, the solvent for chloroquine. Culture medium and treatment were refreshed every two days.

### 2.3. RNA Extraction and RNA-Seq Library Construction and Sequencing

Total RNA from C2C12 cells was extracted using TRIzol reagent and the Direct-zol RNA Microprep kit, according to the suppliers’ instructions. RNA concentration and purity were measured with a Nanodrop Spectrophotometer (Thermo Fisher Scientific). RNA integrity was determined using an Agilent 2100 Bioanalyzer (Agilent Technologies, Santa Clara, CA, USA). All RNA samples for RNA-seq library preparation had RNA integrity numbers (RINs) greater than 7.7. RNA-seq libraries were prepared using the NEBNext Ultra II Directional RNA Library Prep Kit for Illumina (E7760S) (New England BioLabs), according to the manufacturer’s instructions. Each library was uniquely indexed. RNA-seq libraries were paired-end sequenced on an Illumina sequencing system at Novogene Corporation (Sacramento, CA, USA).

### 2.4. Gene Expression Analysis and Gene Ontology Analysis of RNA-Seq Data

The raw sequencing reads were first filtered with Trimmomatic [34] to remove low-quality reads and reads containing adapters. Clean reads were mapped to the mouse reference genome (mm10) using the STAR (v2.5) program [35]. The uniquely mapped reads were used in the quantification of gene expression levels, which was performed using the HTSeq v0.6.1 program [36]. Gene expression levels were calculated as reads per kilobase of exon model per million mapped reads (FPKM) [37]. A differential expression analysis was performed using the DESeq2 R package (2_1.6.3) [38], and the resulting *p*-values were adjusted using the Benjamini–Hochberg approach for controlling the false discovery rate (FDR). Genes with adjusted *p*-values (*P*adj) < 0.05 were considered as differentially expressed genes (DEGs). Gene ontology (GO) analysis of DEGs was performed in three categories: biological process (BP), cellular compound (CC), and molecular function (MF), using the R package clusterProfiler [39,40]. In this analysis, the *p*-value and *q*-value cutoffs were 0.01 and 0.05, respectively.

### 2.5. Reverse Transcription Quantitative PCR (RT-qPCR)

One microgram of total RNA was denatured by incubation with random primers at 70 °C for 10 min, followed by cooling on ice for 5 min. Reverse transcription of total RNA was performed by incubating 5 μL RNA–random primer mix with 4 μL 5 × reverse transcription buffer, 4.8 μL MgCl_2_, 1 μL dNTP, 1 μL ImProm-II reverse transcriptase, 0.5 μL RNasin Ribonuclease Inhibitor, and 3.7 μL RNase free H_2_O at 42 °C for 90 min, at 70 °C for 10 min, and then at 4 °C for 5 min. Quantitative PCR of 20 ng cDNA was performed in duplicate using Fast SYBR Green Master Mix on a 7500 Fast Real-Time PCR system (Applied Biosystems). The PCR primers used in this study were designed in a previous study [41]. Relative gene expression levels were calculated using the ∆∆Ct method [42], using the HMBS gene as a reference gene, which, based on its Ct value (data not shown), was stably expressed in C2C12 cells during differentiation.

### 2.6. Immunocytochemistry

C2C12 cells were first fixed by incubating them with 4% formaldehyde solution for 15 min at room temperature and then rinsed twice with PBS. Cells were then permeabilized by incubation with 0.25% Triton X-100 for 10 min at room temperature. After that, cells were rinsed again with PBS and then incubated with 1% BSA and 0.05% Tween-20 in PBS for one hour at room temperature with shaking to block nonspecific antibody binding. Cells were then incubated with the antibody for myosin heavy chains (NA-4, DSHB, Iowa City, IA, USA) at 1:100 in PBS at 4 °C overnight. Cells were rinsed twice with PBS and incubated with an anti-mouse IgG FITC antibody (Sigma-Aldrich) at 1:200 dilution for 1 h at room temperature. Nuclei were stained by incubating cells with 1 μg/mL of 4′,6-diamidino-2-phenylindole (DAPI) for 10 min at room temperature. Images of stained cells were taken with a florescence microscope.

### 2.7. Determination of Fusion Index, Myotube Length, and Myotube Area

Numbers of nuclei were counted and lengths of myotubes and areas of myotubes were measured from randomly taken immunofluorescent images of myotubes using ImageJ [43]. A myotube was defined as a myosin-heavy-chain-positive (“green”) cell containing 2 or more DAPI-stained nuclei (“blue”); the length of a myotube was defined as the longest distance between two ends of a myotube; and fusion index was defined as the ratio of the number of nuclei in myotubes to the number of total nuclei counted. Around 1000 total nuclei were counted, and 15 myotubes were measured for each treatment in each experiment.

### 2.8. Total Cellular Protein Extraction and Western Blot Analysis

Total protein lysates from C2C12 cells were prepared by scraping and incubating them in the RIPA buffer with proteinase inhibitors added for 30 min on ice followed by centrifugation at 16,000× *g*, 4 °C, for 15 min. Protein concentrations were determined using a Pierce BCA Protein Assay Kit (23227, obtained from Thermo Fisher Scientific). For the Western blot analysis, 20 μg of total protein lysates were separated using sodium dodecyl sulfate–polyacrylamide gel electrophoresis (SDS-PAGE) with 6% stacking and 15% separating gel. Gel electrophoresis was run at 80 V for 30 min and then 120 V for 80 min at 4 °C. Following separation, protein was transferred from the gel to a nitrocellulose membrane by electrophoresis at 70 V, 4 °C, for 60 min. To block nonspecific antibody binding, the membrane was incubated with 5% non-fat milk in the TBST buffer for 1 h at room temperature. The membrane was then incubated with a primary antibody overnight at 4 °C. The membrane was rinsed twice with TBST and then incubated with a secondary antibody for 1 h at room temperature. The following primary antibodies were used in Western blot analyses: ß-tubulin at 1:1000 dilution (E7 from DSHB), LC3B at 1:1000 dilution (NB100-2220 from Novus Biologicals, Centennial, CO, USA), and myogenin at 1:25 dilution (F5D from DSHB). The following secondary antibodies were used in Western blot analyses: IRDye 800CW goat anti-rabbit IgG secondary antibody (926-32211, LI-COR Biosciences, Lincoln, NE, USA) at 1:15000 dilution and IRDye 800CW goat anti-mouse IgG secondary antibody (926-32210, LI-COR Biosciences) at 1:15,000 dilution. Western blots were visualized with the LI-COR Odyssey Infrared Image System 9120, and the fluorescent intensities were quantified with the Image Studio Lite software (LI-COR Biosciences).

### 2.9. Statistical Analyses

Data were analyzed by ANOVA, followed by multiple comparisons using Tukey’s test or Dunnett’s test. Two-group comparisons were carried out via *t*-tests. All statistical analyses were performed in JMP Pro 16 (SAS, Cary, NC, USA) or GraphPad Prism 9 (San Diego, CA, USA). All data are expressed as means ± standard errors.

## 3. Results

### 3.1. More Than Ten Thousand Genes Were Differentially Expressed during Myoblast Differentiation

**Figure 1:**
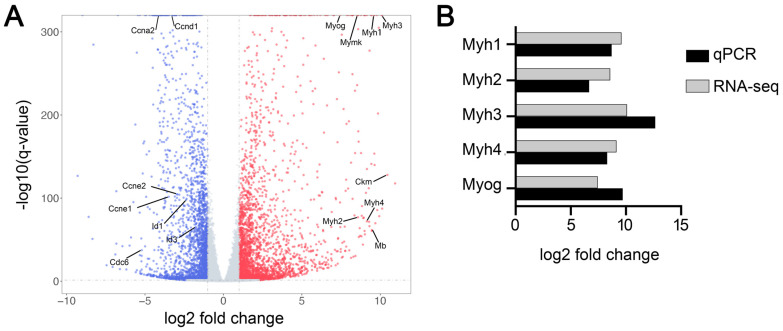
Identification of differentially expressed genes during myoblast differentiation. mRNA transcriptomes in C2C12 cells before (*n* = 5) and 6 days after (*n* = 5) induction of myogenic differentiation were analyzed by RNA-seq. (**A**) The overall distribution of differentially expressed genes (*P*adj < 0.05). Genes upregulated and downregulated on day 6 of differentiation compared to before differentiation are indicated by red and blue dots, respectively. (**B**) Comparison of the fold changes in the expression of selected mRNAs determined by RNA-seq and RT-qPCR.

We performed an RNA-seq analysis to identify genes differentially expressed during myogenic differentiation in C2C12 cells. The sequencing of five RNA-seq libraries constructed from C2C12 cells immediately before induction of myogenic differentiation and five RNA-seq libraries constructed from C2C12 cells on day 6 of induced myogenic differentiation generated more than 36 million unique mapped sequencing reads per library (Supplementary Table S1). Gene expression quantification and subsequent differentiation expression analysis revealed 11,046 genes differentially expressed (*P*adj < 0.05) between the two conditions, of which 5615 genes were upregulated and 5431 genes downregulated from the day before induction of differentiation to day 6 of differentiation (Figure 1A, Supplementary Table S2). Examples of genes upregulated in C2C12 cells during induced myogenic differentiation were *Myh1*, *Myh2*, *Myh3*, *Myh4*, *Myog, Ckm*, *Mymk*, and *Mb,* which are all known to be specifically or preferentially expressed in skeletal muscle [44,45]. Examples of genes downregulated during this differentiation included *Ccna2*, *Ccnd1*, *Ccne1*, *Ccne2*, *Id1*, *Id3*, and *Cdc6*, which are known to function in cell proliferation [46,47]. To confirm the gene expression differences quantified by RNA-seq, we measured the expression levels of *Myh1*, *Myh2*, *Myh3*, *Myh4*, and *Myog* mRNAs in the two conditions by RT-qPCR. As shown in Figure 1B, average fold changes measured by RT-qPCR were similar to those quantified by RNA-seq for all of these mRNAs.

### 3.2. Autophagy Was Upregulated during Myoblast Differentiation

**Figure 2:**
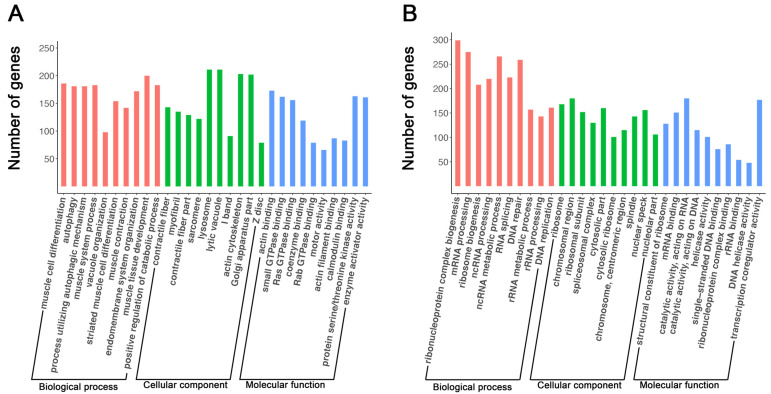
Top 10 biological processes, cellular components, and molecular functions associated with genes upregulated (**A**) and downregulated (**B**) during myoblast differentiation. The *X*-axis indicates the functional terms, and the *Y*-axis indicates the number of genes linked to each functional term. In each functional category, terms are ranked from left to right according to their adjusted *p*-values, with the leftmost term having the smallest *p*-value.

Gene ontology enrichment analyses of genes upregulated during myogenic differentiation in C2C12 cells revealed the biological processes (BPs), cellular components (CCs), and molecular functions (MFs) associated with these genes (Supplementary Table S3). Shown in Figure 2A are the top 10 upregulated BPs, CCs, and MFs during myogenic differentiation in C2C12 cells. Shown in Table 1 are two examples of top upregulated BPs and associated genes. As expected, most of these upregulated BPs were related to skeletal muscle development and maturation, including muscle cell differentiation, muscle system process, striated muscle cell differentiation, muscle contraction, and muscle tissue development (Figure 2A). Interestingly, four of the top upregulated BPs were related to autophagy (Figure 2A, Table 1). Similar to the BPs, most of the top upregulated CCs during myogenic differentiation in C2C12 cells were related to skeletal muscle structures, such as sarcomeres, I-bands, and Z-discs (Figure 2A). Two of the top upregulated CCs were related to organelles that are involved in protein degradation, namely, lysosomes and lytic vacuoles (Figure 2A). The top upregulated MFs during myogenic differentiation in C2C12 cells included actin binding, motor activity, and calmodulin binding, which are all known as functions of mature skeletal muscle [48,49]. It is also interesting to note that small GTPase binding, Ras GTPase binding, coenzyme binding, Rab GTPase binding, protein serine/threonine kinase activity, and enzyme activator activity were among the MFs upregulated during myogenic differentiation in C2C12 cells (Figure 2A).

GO analyses of genes downregulated during myogenic differentiation in C2C12 cells indicated that many of them were associated with BPs, CCs, and MFs related to DNA replication and RNA processing (Supplementary Table S4). Examples of the top downregulated BPs during myogenic differentiation in C2C12 cells were ribonucleoprotein complex biogenesis and DNA replication; examples of top downregulated CCs were ribosomes and chromosomal regions; and examples of top downregulated MFs were mRNA binding and helicase activity (Figure 2B, Table 2).

### 3.3. Myoblast Differentiation Was Associated with Increased Conversion of LC3-I to LC3-II

**Figure 3:**
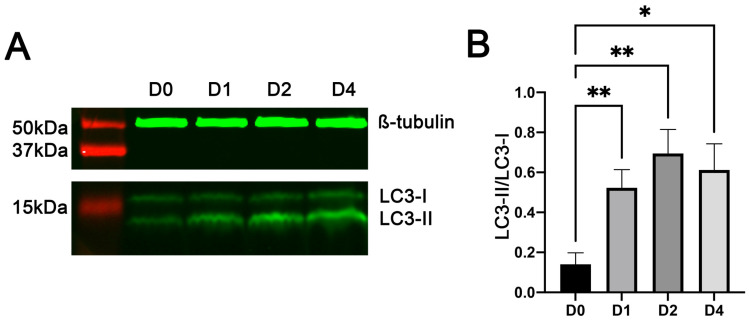
Increased generation and accumulation of LC3-II in C2C12 cells during myogenic differentiation. Total protein lysates from C2C12 cells on day (D) 0 (i.e., before differentiation) and days 1, 2, and 4 of myogenic differentiation were analyzed by Western blotting. (**A**) Representative images of Western blots. (**B**) Average ratios of LC3-II to LC3-I protein. * and ** indicate *p* < 0.05 and <0.002, respectively (*n* = 6), based on Dunnett’s test.

LC3 protein, encoded by the microtubule-associated proteins 1A/1B light chain 3B (*Map1lc3b*) gene, is the most widely used marker of autophagy [50]. During autophagy, the cytosolic LC3 protein LC3-I is conjugated to phosphatidylethanolamine to form the autophagosomal membrane protein LC3-II [51]. Western blot analyses (Figure 3A) showed that the protein expression ratio of LC3-II to LC3-I in C2C12 cells increased rapidly from the day before induction of differentiation (day 0 in the figure) to day 1 of differentiation and remained high on day 2 and day 4 of differentiation (Figure 3B). These changes indicated that the autophagic flux increased in C2C12 cells during myogenic differentiation, confirming what was revealed by the RNA-seq analysis described above.

### 3.4. Inhibition of Autophagy Reduced the Number, Length, and Size of Myotubes Formed during Myoblast Differentiation

**Figure 4:**
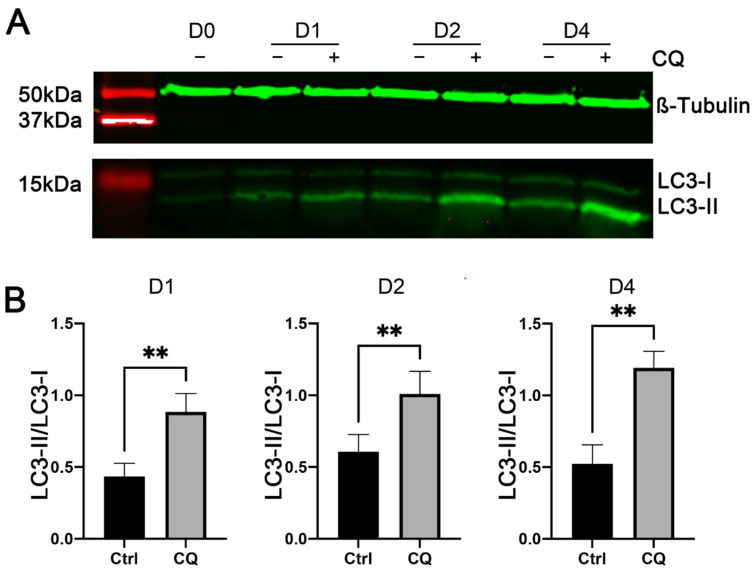
Effects of chloroquine (CQ) on LC3-I and LC3-II protein expression in C2C12 cells during myogenic differentiation. C2C12 cells were cultured in differentiation medium in the presence (+) or absence (−) of 20 μM CQ. Total protein lysates from cells on days 0 (D0, before differentiation), 1 (D1), 2 (D2), and 4 (D4) of differentiation were analyzed by Western blotting, using antibodies specific to LC3 and β-Tubulin (as a loading control). (**A**) Representative images of Western blots. (**B**) Ratio of LC3-II to LC3-I protein. ** indicates *p* < 0.002 (*n* = 5), based on *t*-tests.

To determine the role of increased autophagy in myoblast differentiation, we induced C2C12 cells to differentiate in the presence of chloroquine (CQ), a widely used inhibitor of autophagic flux that inhibits autophagy by blocking the fusion of autophagosomes with lysosomes [32]. We first performed Western blot analyses to determine whether CQ is effective in inhibiting autophagy in C2C12 cells. As shown in Figure 4A,B, the ratios of LC3-II to LC3-I in C2C12 cells treated with CQ were significantly higher than those in control C2C12 cells on days 1, 2, and 4 of myogenic differentiation. These increases confirmed the effectiveness of CQ in blocking autophagosome–lysosome fusion and hence the degradation of LC3-II protein by the lysosomal hydrolases. These Western blot analyses also confirmed the increases in the generation of LC3-II in C2C12 cells during myogenic differentiation (Figure 4A).

**Figure 5:**
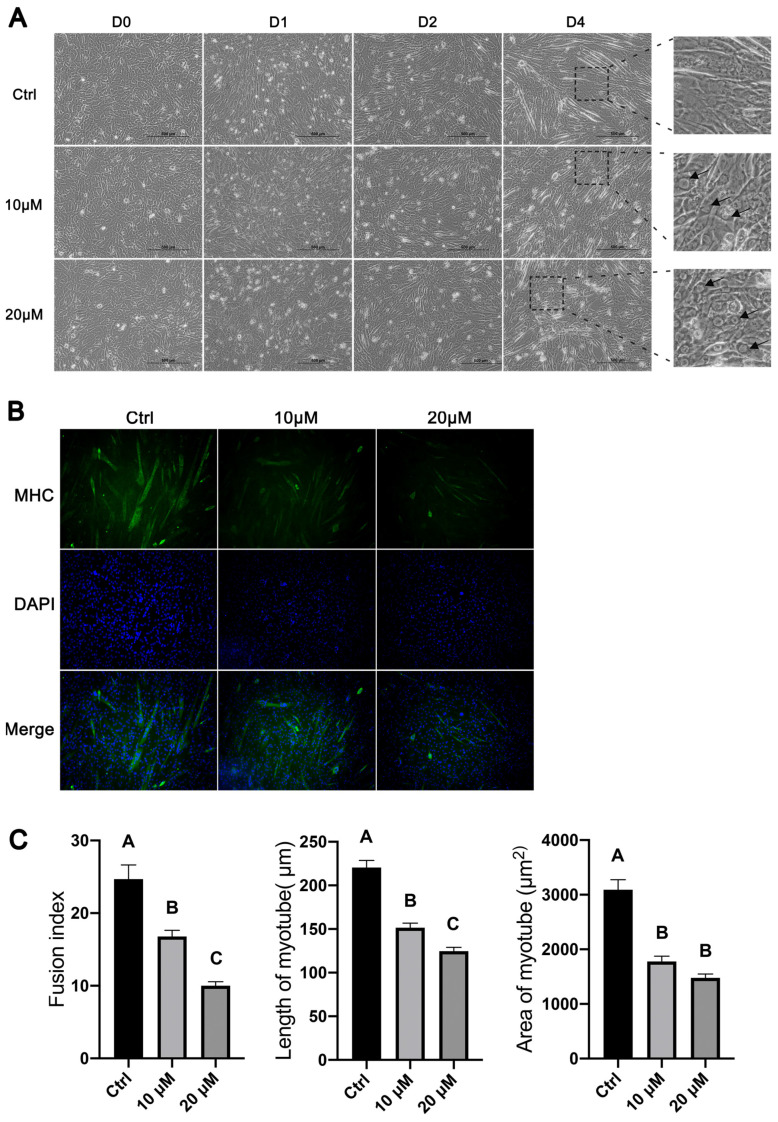
Effects of inhibiting autophagy with chloroquine on the morphological changes in C2C12 cells during myoblast differentiation. C2C12 cells were cultured in differentiation medium in the presence of 10 or 20 μM chloroquine. Control (ctrl) C2C12 cells were cultured in differentiation medium in the absence of chloroquine. (**A**) Representative micrographs of C2C12 cells on days (D) 0, 1, 2, and 4 of differentiation. Arrows point to “dark rings” around the nuclei in CQ-treated cells. (**B**) Representative micrographs of C2C12 cells on day 4 of differentiation. Cells were stained with myosin heavy-chain (MHC) antibody (green) and DAPI (blue). (**C**) Quantification of fusion index and length and area of myotubes. Bars not sharing the same letter labels are different (*p* < 0.05, *n* = 8), based on Tukey’s test.

Based on the morphology, multinucleated myotubes began to form in C2C12 cells two days after the initiation of myogenic differentiation, and multinucleated myotubes could be easily observed by day 4 of differentiation (Figure 5A). C2C12 cells treated with CQ appeared to form fewer and smaller myotubes than untreated C2C12 cells on the same day of differentiation (Figure 5A). The higher concentration (20 μM) of CQ apparently caused greater decreases in the number and size of formed myotubes than the lower concentration (10 μM) of CQ; myotubes were barely seen in C2C12 cells treated with 20 μM CQ on day 4 of differentiation (Figure 5A). Interestingly, at high magnification, “dark rings” around the nuclei could be observed in CQ-treated C2C12 cells (Figure 5A). These “dark rings” were probably formed by autophagosomes which accumulated due to their blocked fusion with lysosomes and hence their blocked degradation by lysosomal enzymes.

To more clearly show the morphological differences between CQ-treated and control C2C12 cells, we stained the myosin heavy-chain proteins and nuclei in C2C12 cells on day 4 of differentiation and quantified the number of nuclei fused into myotubes and the lengths and areas of myotubes. As shown in Figure 5B, myotubes in CQ-treated C2C12 cells were clearly fewer and smaller than in control C2C12 cells, and these differences were more obvious with the higher concentrations of CQ. The average fusion indexes of C2C12 cells treated with 10 μM and 20 μM CQ were approximately 30% and 60%, respectively, lower than that of control C2C12 cells (*p* < 0.05, Figure 5C). The average lengths of myotubes formed from C2C12 cells treated with 10 μM and 20 μM CQ were approximately 31% and 43%, respectively, shorter than that of myotubes formed from control C2C12 cells (*p* < 0.05, Figure 5C). The average areas of myotubes formed from C2C12 cells treated with 10 μM and 20 μM CQ were 40% and 50%, respectively, smaller than that of myotubes formed from control C2C12 cells (*p* < 0.05, Figure 5C).

### 3.5. Inhibition of Autophagy Reduced the Expression of Muscle-Specific Genes during Myoblast Differentiation

**Figure 6:**
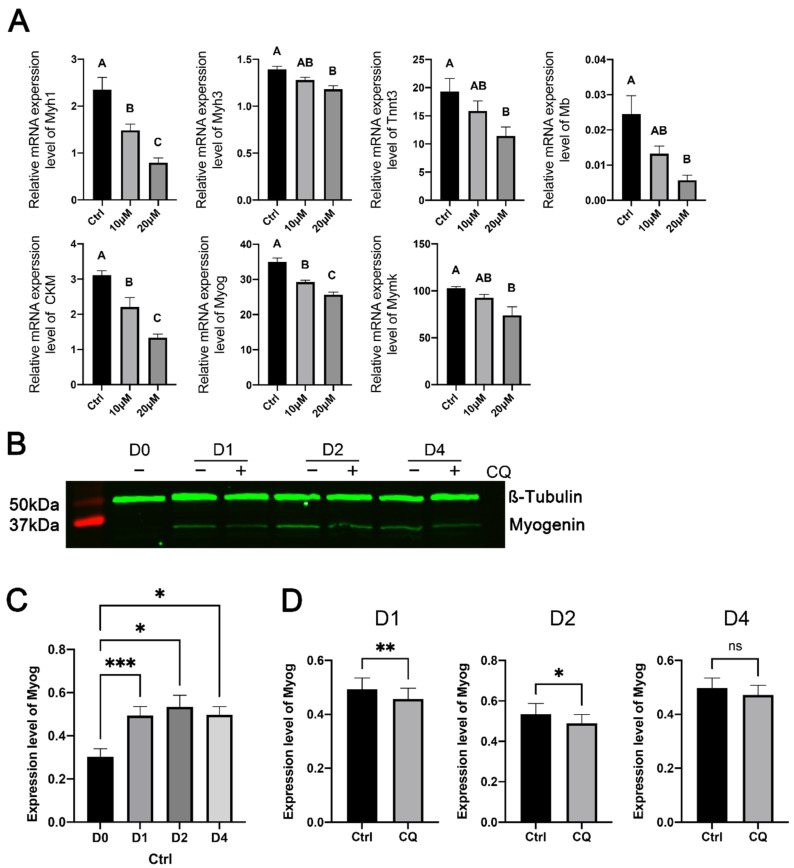
Effects of inhibiting autophagy with chloroquine on the expression of selected muscle-specific genes in C2C12 cells during myogenic differentiation. C2C12 cells were cultured in differentiation medium in the presence of 10 or 20 μM chloroquine for 4 days. Control (ctrl) C2C12 cells were cultured in differentiation medium without chloroquine. (**A**) Relative mRNA expression levels. Bars not sharing the same letter label are different (*p* < 0.05, *n* = 6), based on Tukey’s test. (**B**) Representative Western blots of myogenin. (**C**) Comparison of myogenin protein expression levels in C2C12 cells between day (D) 0 and days 1, 2, and 4 of differentiation. * and *** indicate *p* < 0.05 and 0.01, respectively, based on Dunnett’s test (*n* = 5). (**D**) Comparison of myogenin protein expression levels in C2C12 cells between chloroquine and control. *, ** and ns indicate *p* < 0.05, 0.002 and >0.05, respectively, based on *t*-tests (*n* = 5). Data were log-transformed for normality prior to statistical analysis.

To further determine the effect of the inhibition of autophagy on myoblast differentiation, we quantified the expression levels of several skeletal-muscle-specific genes in CQ-treated and control C2C12 cells on day 4 of myogenic differentiation. These genes included *Myh1*, *Myh3*, *Tnnt3*, *Mb*, *Ckm*, *Myog*, and *Mymk,* which encode either structural or functional components of skeletal muscle (*Myh1*, *Myh3*, *Tnnt3*, *Mb*, and *Ckm*) or are master regulators of myoblast terminal differentiation and fusion (*Myog* and *Mymk*) [44]. As shown in Figure 6A, the expression of most of these genes was inhibited in C2C12 cells treated with 10 μM CQ, and the expression of all of these genes was inhibited in C2C12 myoblasts treated with 20 μM CQ compared with their expression in untreated C2C12 cells.

We also confirmed the effect of the inhibition of autophagy on the expression of myogenin at the protein level. As expected, myogenin protein expression in untreated C2C12 cells was significantly higher on days 1, 2, and 4 of differentiation than on the day before the induction of differentiation (Figure 6B,C). Compared to the control, CQ significantly reduced myogenin protein expression in C2C12 cells on days 1 and 2 of differentiation (Figure 6D).

## 4. Discussion

In this study, we first compared the gene expression profiles between undifferentiated and differentiating C2C12 myoblasts. Our RNA-seq analysis revealed that the expression of many skeletal-muscle-specific genes increased while the expression of many genes involved in the cell cycle decreased during myoblast differentiation. These results were consistent with those of earlier microarray-based gene expression profiles for C2C12 cells [52,53,54], indicating that myoblast differentiation involves a shift in gene expression from genes functioning in cell proliferation to genes functioning in skeletal muscle buildup and contraction. Our RNA-seq analysis revealed that many genes functioning in mRNA processing, ribonucleoprotein complex biogenesis, and ribosomes were downregulated during myogenic differentiation in C2C12 cells. This result suggests that myoblast differentiation is associated with delayed processing (i.e., capping, polyadenylation, and splicing) of newly synthesized mRNA (i.e., pre-mRNA) in the nucleus, delayed export of processed mRNA from the nucleus to the cytoplasm, and delayed translation of exported mRNA in the cytoplasm. These delays may be meant to reduce loss of mRNAs through membrane pores formed during myoblast fusion [55].

Our RNA-seq analysis also revealed that many genes involved in autophagy and related functional terms were upregulated during myoblast differentiation. These gene expression changes as well as those involved in ribonucleoprotein complex biogenesis and mRNA processing mentioned above were not identified by earlier microarray-based gene expression analyses [52,53,54], perhaps because many of the genes involved in autophagy, ribonucleoprotein complex biogenesis, and mRNA processing were not included in the microarrays or in the functional analyses performed in these studies, or because microarray-based gene expression analysis is less sensitive than RNA-seq in detecting changes in gene expression, in particular, changes in low-abundance transcripts [56,57,58].

The upregulation of genes functioning in autophagy during myoblast differentiation indicates that autophagy-mediated protein degradation increases during myoblast differentiation. Indeed, we confirmed increased autophagy in C2C12 cells during myogenic differentiation by detecting increased conversion of LC3-I to LC3-II in differentiating C2C12 myoblasts and by detecting increased accumulation of LC3-II in autophagy-blocked differentiating C2C12 myoblasts. The association of increased autophagy with myoblast differentiation suggests that autophagy might benefit myoblast differentiation. A positive role for autophagy in myoblast differentiation is supported by our results showing that blocking autophagy in C2C12 myoblasts impaired their expression of muscle-specific genes and their fusion into multinucleated myotubes. Our finding that autophagy plays a positive role in myoblast differentiation was consistent with earlier studies using different approaches or different myoblast models [24,25,26,27,28,29]. However, the conclusion that autophagy aids myoblast differentiation seems to contradict the observation that inducing autophagy with the mTOR inhibitor rapamycin inhibited myoblast differentiation in C2C12 cells [59]. One possible reason for this discrepancy is that the target of rapamycin, mTOR, controls not only autophagy but also other processes, such as overall protein synthesis, which is essential for myoblast differentiation [60,61].

Autophagy is a process by which cells maintain hemostasis and survival by delivering organelles and proteins to lysosomes for degradation [62]. There is likely increased generation of damaged, malformed, or unnecessary organelles and proteins during the differentiation and fusion of mononuclear myoblasts into multinucleated myotubes [63]. Therefore, increased autophagy may be one of the means used by myoblasts to clear these organelles and proteins and thereby maintain normal myogenic differentiation. Indeed, this role for autophagy in myoblast differentiation is supported by previous findings that upregulated autophagy is essential for mitochondrial degradation and biogenesis and protection against mitochondrial oxidative stress during myoblast differentiation [64,65].

Myogenin is a master transcriptional regulator of myoblast differentiation [8]. Myogenin binds to and activates the transcription of muscle-specific genes as a heterodimer with E-proteins [66]. However, the DNA-binding and transactivating ability of myogenin is inhibited by a group of proteins called the inhibitor of DNA binding (ID) proteins, which lack basic DNA binding domains [66]. The ubiquitin–proteasome system was previously found to degrade the ID proteins in differentiating myoblasts [67]. We speculate that the autophagy system might also degrade the ID proteins and thereby promote myoblast differentiation. Besides the ID proteins, proteins such as the cyclin-dependent kinases and transformation growth factor beta (TGF-ß) also inhibit myoblast differentiation by keeping myoblasts in the proliferation phase [46,68]. We speculate that the autophagy system could also target and degrade these proteins to promote myoblast differentiation.

## 5. Conclusions

A massive number of genes are upregulated or downregulated during myoblast differentiation. Genes upregulated during myoblast differentiation include those associated with skeletal muscle structure and contraction and autophagy. Genes downregulated during myoblast differentiation include those involved in the cell cycle, ribonucleoprotein complex biogenesis, and mRNA processing. Not only the expression of autophagic genes but autophagic activity are increased during myoblast differentiation. Increased autophagy is confirmed to benefit myoblast differentiation and fusion.

## Figures and Tables

**Table 1:** Two examples of functional terms associated with genes upregulated during myoblast differentiation.

ID	Description	Gene Ratio	*p*-Value	GeneID
GO:0042692	muscle cell differentiation	186/5118	1.28 × 10^−28^	*Pgm5*, *Myl2*, *Cacna1s*, *Lmod2*, *Tnnt3*, *Mybpc2*, *Myoz2*, *Mybpc1*, *Actc1*, *Casq1*, *Trim72*, *Actn2*, *Neb*, *Casq2*, *Myom3*, *Myh6*, *Cav3*, *Ankrd2*, *Csrp3*, *Mypn*, *Tcap*, *Rbm24*, *Klhl41*, *Tnnt1*, *Acta1*, *Myom1*, *Myom2*, *Fbxo40*, *Lmod3*, *Igf2*, *Igf1*, *Dmd*, *Myog*, *Alpk3*, *Ins2*, *Klhl40*, *Ryr1*, *Ankrd23*, *Tmod1*, *Myo18b*, *Ttn*, *Sypl2*, *Mef2c*, *Neu2*, *Smyd1*, *Synpo2l*, *Hopx*, *Nrap*, *Dysf*, *Fgf9*, et al.
GO:0006914	autophagy	181/5118	1.32 × 10^−27^	*Dcn*, *Fez1*, *Mapt*, *Trem2*, *Synpo2*, *Epm2a*, *Bmf*, *Htr2b*, *Casp1*, *Prkaa2*, *Trp53inp2*, *Ifng*, *Pink1*, *Lzts1*, *Map1lc3a*, *Pik3c2b*, *Srpx*, *Rnf152*, *Rasip1*, *Mtm1*, *Tlr2*, *Irgm2*, *Lix1*, *Fbxw7*, *Usp13*, *Snapin*, *Adrb2*, *Fyco1*, *Trp53inp1*, *Stat3*, *Atp6v0a1*, *Nupr1*, *Tfeb*, *Dram2*, *Trib3*, *Nod1*, *A tg4a*, *Ctsd*, *Qsox1*, *Atg10*, *Zfyve1*, *Trim13*, *Atg13*, *Vps13d*, *Flcn*, *Wipi1*, *Nod2*, *Fbxl2*, *Sirt2*, *Hspb8*, et al.

**Table 2:** Two examples of functional terms associated with genes downregulated during myoblast differentiation.

ID	Description	Gene Ratio	*p*-Value	GeneID
GO:0022613	ribonucleoprotein complex biogenesis	299/4657	1.47 × 10^−107^	*Celf4*, *Suv39h1*, *Lyar*, *Exosc8*, *Nop56*, *Lsm2*, *Npm1*, *Lsm3*, *Dkc1*, *Nhp2*, *Fbl*, *Gar1*, *Gemin6*, *Snrpd1*, *Dis3*, *Ppan*, *Ruvbl2*, *Hsp90aa1*, *Nop2*, *Ncl*, *Ran*, *Noc2l*, *Pa2g4*, *Exosc2*, *Xpo1*, *Nop58*, *Rcl1*, *Snrpg*, *Npm3*, *Rrp15*, *Snrpe*, *Rbmxl1*, *Mybbp1a*, *Ddx20*, *Ddx18*, *Rrp9*, *Ruvbl1*, *Ddx21*, *Rrs1*, *Snrpd3*, *Mrto4*, *Utp20*, *Rpsa*, *Eri1*, *Lsm6*, *Gemin5*, *Lsm4*, *Srsf1*, *Wdr43*, *Rps2*, et al.
GO:0006397	mRNA processing	275/4657	5.32 × 10^−83^	*Ccnb1*, *Celf4*, *Hmx2*, *Ptbp1*, *Lsm2*, *Slbp*, *Npm1*, *Lsm3*, *Hnrnpa1*, *Lsm5*, *Snrpa1*, *Gemin6*, *Snrpd1*, *Ppil1*, *Rbmx2*, *Alyref*, *Pabpc1*, *Mbnl3*, *Khdrbs3*, *Adarb1*, *Srsf7*, *Papolb*, *Ddx39*, *Snrpg*, *Zfp473*, *Srsf9*, *Dazap1*, *Lsm8*, *Tbrg4*, *Snrnp40*, *Magohb*, *Snrpe*, *Rbmxl1*, *Ddx20*, *Srrt*, *U2af2*, *Lsm7*, *Snrpd3*, *Hnrnph1*, *Hnrnpm*, *Tra2a*, *Ttf2*, *Hnrnpa3*, *Snrpa*, *Rbm19*, *Cstf2*, *U2af1*, *Lsm6*, *Gemin5*, *Lsm4*, et al.

## Data Availability

The sequencing data from this study has been deposited in the NCBI GEO database (https://www.ncbi.nlm.nih.gov/geo/) under accession number GSE215627.

## Supplementary Materials

The following supporting information can be downloaded at: https://www.mdpi.com/article/10.3390/cells11223549/s1, Table S1: Summary of RNA sequencing and mapping results; Table S2: List of all differentially expressed genes; Table S3: Biological processes, cellular components, and molecular functions associated with upregulated genes; Table S4: Biological processes, cellular components, and molecular functions associated with downregulated genes.
