# Supplementary material for: RNA-Sequencing Reveals Upregulation and a Beneficial Role of Autophagy in Myoblast Differentiation and Fusion
Source: Cells. 2022 Nov 10;11(22):3549. doi: 10.3390/cells11223549 (PMC9688917; doi:10.3390/cells11223549)
Supplement: Supplementary file 1 [file cells-11-03549-s001.zip › cells-2001025-supplementary Table S1.pdf]

Table S1. Summary of RNA sequencing and mapping results.

RNA-seq Library	Raw Reads	Trimmed Reads	Total Mapped Reads	Total Mapping Rate	Uniquely Mapping Rate	Uniquely Mapped Reads
C2A1	48893870	47432376	43818358	92.38%	41821598	88.17%
C2A2	51693558	50472662	45899728	90.94%	43894184	86.97%
C2A3	59952808	58406158	53085534	90.89%	50848798	87.06%
C2A4	42381668	41385146	38635612	93.35%	36969986	89.33%
C2A5	43625236	42267268	39524602	93.51%	37748072	89.31%
C2B1	56319126	55044342	50614278	91.95%	48421854	87.97%
C2B2	52221498	50978384	46700452	91.61%	44836580	87.95%
C2B3	45035416	43807398	40159780	91.67%	38608598	88.13%
C2B4	45496936	44365806	40480144	91.24%	38874062	87.62%
C2B5	42239522	41160068	37778122	91.79%	36293690	88.18%
